# An actionable roadmap for NTD morbidity, disability, and disfigurement management

**DOI:** 10.1371/journal.pntd.0013760

**Published:** 2025-12-08

**Authors:** Francisca Mutapi, Ibrahima Socé Fall, Humphrey D. Mazigo, Paradzayi Tagwireyi, Hassan Ahmed Hassan Ahmed Ismael, Geoffrey Banda, Paul Erasto Kazyoba, Takafira Mduluza

**Affiliations:** 1 Institute of Immunology and Infection Research, University of Edinburgh, Ashworth Laboratories, King’s Buildings, Edinburgh, United Kingdom; 2 Tackling Infections to Benefit Africa, University of Edinburgh, Ashworth Laboratories, King’s Buildings, Edinburgh, United Kingdom; 3 Department of the Control of Neglected Tropical Diseases, World Health Organization, Geneva, Switzerland; 4 Department of Medical Parasitology, School of Medicine, Catholic University of Health, and Allied Sciences, Mwanza, Tanzania; 5 Department of Geography and Environmental Science, Geo-information and Earth Observation Centre, University of Zimbabwe, Mount Pleasant, Harare, Zimbabwe; 6 Biomedical Research Institute, Ibn Sina University, Soba Campus, Khartoum, Sudan; 7 The Innogen Institute, University of Edinburgh, Science Technology and Innovation Studies, Edinburgh, United Kingdom; 8 Department of Research and Development, National Institute for Medical Research, Dar-Es-Salaam, Tanzania; 9 Department of Biochemistry, University of Zimbabwe, Mount Pleasant, Harare, Zimbabwe; Federal University of Ceará, Fortaleza, BRAZIL

## Abstract

Significant progress has been made in controlling neglected tropical diseases (NTDs). As of July 2025, 57 countries have eliminated at least one NTD as a public health problem or with interruption of transmission. Nonetheless, progress has not been uniform across the spectrum of NTD health service delivery. Comparatively, less progress has been made in managing chronic disease. Less than 15% of the 178 NTD-endemic countries currently have guidelines, protocols, or policies dedicated to the management of disabilities and secondary health problems resulting from widespread NTDs in their countries. To provide comprehensive health services to people living with chronic NTD diseases, there is a need to understand the impact of these diseases on the daily lives of affected people, health system shortcomings in providing treatment and management, and policy gaps. As part of a larger study, we conducted inclusive consultative workshops involving people suffering from NTD-related chronic diseases, disability, and disfigurement, health workers, policy makers, and NTDs researchers from Sudan, Tanzania, and Zimbabwe. Through this, we co-produced an NTD-affected voices-led roadmap to inform health service provision for NTD-affected people. The roadmap made 5 calls to action directed at different groups: affected voices, health workers, policy makers, ministries of health, and development partners. Responding to these calls through the actionable roadmap will improve the management of NTD-related disease including complications, disfigurement, and disability.

## Author Summary

The workshop participants made the following five calls to action to ministries of health and development partners:

- Ministries of health must develop guidelines, protocols, and policies for managing neglected tropical diseases (NTD)-related disabilities, disfigurement, and mental health.
- The NTD-affected voices movement needs to be institutionalized at all levels of decision-making and health service provision, including grassroots participants, NGOs, ministries of health, and development partners.
- Effective communication and advocacy are needed to ensure accurate and targeted health education and the success of awareness campaigns. This should be complemented by community engagement to increase health-seeking behavior, intervention uptake, and trust between the affected voices and health service providers.
- Equip and strengthen health systems to respond to mental health challenges, disfigurement, and disabilities brought on by NTDs.
- Researchers and innovators must continue to work on improved diagnostics, therapeutics, and interventions, and funders must fund this work.

## Introduction

Neglected tropical diseases (NTDs) are a diverse group of 21 disabling and debilitating conditions that affect an estimated 1.5 billion people. The most severely impacted communities tend to be the vulnerable and the marginalized, with limited access to essential resources like safe water, sanitation, and healthcare. These diseases not only cause severe debilitation and mortality but also perpetuate cycles of poverty, imposing substantial economic costs on affected nations.

Recent initiatives such as the Kigali Declaration [1], and the World Health Organization (WHO)’s new NTD road map for 2021–2030 [2] among others have brought renewed focus to NTDs, emphasizing the need for multi-stakeholder collaboration, innovative approaches, and heightened political commitment [2]. The WHO 2024 Global report on neglected tropical diseases reports that significant progress has been made towards meeting the 2030 targets set in 2020 [3]. For example, as of July 2025, 57 countries have eliminated at least one NTD [4]. This is more than halfway towards the road map’s target of 100 countries eliminating at least one NTD by 2030—a milestone reached before the midway point of the target date.

However, this progress has not been distributed equally among people affected by NTDs, as the focus has disproportionately been on treating infection with less emphasis on morbidity and chronic disease management. The large number of individuals living with chronic and debilitating manifestations of NTDs often remains overlooked by national control programs, further exacerbating their suffering and hindering effective management of these diseases. The burden of NTD-related disfigurement and disability is high. For example, 2.4 million people in sub-Saharan Africa suffer from epilepsy related to tapeworm infection [5]. This infection is responsible for 30–70% of the epilepsy cases in areas endemic for tapeworms [6]. While there are treatments and surgical procedures to reduce seizures, these are often not available to sufferers in endemic countries. Over 35 million people suffer from swelling of organs (e.g., limbs, testicles in males) due to elephantiasis [7], and just under 2 million people globally have lost their vision or been visually impaired due to trachoma [8]. Schistosomiasis causes several chronic conditions including vomiting blood, loss of consciousness due to bleeding, esophageal varices [9], liver fibrosis and urinary bladder cancer [10], and enlarged liver and spleen [11].

Studies in Uganda show that delivering appropriate health services is currently compromised by lack of data on the scale of the problem as well as details on who is affected [12]. Research in several African countries (e.g., Kenya, Uganda, and Ethiopia) has highlighted the profound socioeconomic and psychological impacts of NTDs on affected individuals and their families particularly for women [13,14]. These few examples demonstrate how addressing the management of NTD-related chronic morbidity, disability, and disfigurement requires comprehensive strategies that integrate healthcare provision, community engagement, and socioeconomic support.

The WHO’s 2024 Global report on neglected tropical diseases [3] highlights the poor progress made in health service delivery to people living with these NTD-related diseases, disabilities, and disfigurements. Of the 178 countries considered endemic for at least one NTD that causes disability, only 19 countries [3] currently have guidelines, protocols, or policies dedicated to the management of disabilities resulting from NTDs widespread in the country. This perpetuates the neglect of millions of people and compromises universal health coverage.

To provide a comprehensive health service that caters to this group of NTD-affected people, there is a need to understand the impact of the diseases on their daily lives. The Global Burden of Disease (GBD) framework quantifies the impact of NTDs through disability-adjusted life years (DALYs), combining years of life lost with years lived with disability derived from different health states [15]. While this approach captures mortality and many forms of morbidity, it does not fully reflect the burden of NTD-related disfigurement, stigma, and psychosocial consequences [16]. For example, conditions such as leprosy and lymphatic filariasis cause visible disfigurements whose social and psychological impacts are poorly represented in DALY calculations [17,18]. This limitation suggests that GBD estimates may underestimate the true societal and individual burden of NTDs.

Understanding the lived experiences of affected people is critical to fully comprehending the challenges arising from the disease and the desired appropriate interventions. The WHO recognizes the critical role of people affected by NTDs, particularly women and girls, persons with disabilities, and minority or underrepresented groups. As such, delivering the third pillar of the new WHO roadmap [2]—i.e., to change the operating models and culture to facilitate country ownership on NTD control programmes—requires engagement of the NTD-affected people. However, this is easier said than done as few countries have structures to engage with these groups effectively.

To reduce the current health inequality faced by people living with the chronic manifestations of NTDs, it is critical to put the affected people at the center of NTD programmes and decision-making processes, starting with consultative engagements. Thus, we conducted inclusive consultative workshops from which we co-produced a roadmap for collective action. The rationale was to convene the different stakeholders with experiences of different sectors of the NTD ecosystem in one room to discuss the management of chronic NTDs—the persons affected directly by the different NTDs, the health workers providing the health services they use, the policy makers that inform NTD control and health policy, national NTD control programme managers, and researchers across several disciplines. The objective was to conduct a study to co-create an actionable roadmap that could contribute to a policy brief for use by ministries of health and stakeholders to inform the management of chronic NTD manifestations.

## Methods

### Ethical statement

The workshops were part of a collaborative study that received ethical approval in Tanzania from the National Ethical Review Committee board (NIMR/HQ/R.8a/Vol.IX/3590 and NIMR/HQ/R.8 C/Vol.I/1973) and in Zimbabwe from the Medical Research Council of Zimbabwe (MRCZ/A/3030). No studies were conducted in Sudan for this workshop and no Sudanese participants beyond the collaborating Principal Investigators took part in the workshops.

This first study is part of a larger project in Zimbabwe and Tanzania which includes a situational analysis (submitted), and documentation of NTD lived experiences. This work amplifies the voices of people with lived experiences of NTDs and aims to ultimately improve the health service provision for people living with chronic NTD conditions. The project focused on the four most prevalent NTDs in both countries that also cause the highest prevalence of disability or disfigurement [19–21].

### Workshops

Within the past 12 months, members of the TIBA Partnership (TIBA Edinburgh, TIBA Sudan, TIBA Tanzania, and TIBA Zimbabwe) arranged two such consultative meetings. Specifically, we convened a 3-day workshop in Zimbabwe and a 2-day workshop in Zanzibar, Tanzania. The first workshop was in Bindura, Mashonaland Province, Zimbabwe in December 2023. Bindura town is located 89 km northeast of the capital city of Zimbabwe (Harare). The town was selected for the workshop as it is within easy reach of NTD-endemic districts, ensuring many affected people could attend the workshop. The second workshop was conducted in Zanzibar in March 2024. Zanzibar was selected for accessibility and its endemicity to lymphatic filariasis and schistosomiasis among other NTDs.

Before participants took part in the workshops, the aims and procedures were explained to them in their local languages (Shona for Zimbabweans and Kiswahili for Tanzanians) after which they were invited to enroll in the project. Formal consent was then obtained in writing from adults (aged 20 years and above). In the case of the two participating children aged 11 and 13, formal written consent was obtained the parent/guardian.

### Workshop objectives

The two workshops had five objectives:

1. To share countries’ experiences in managing morbidities and disabilities and how the affected are engaged in this aspect.
2. To identify gaps and bottlenecks that affect the delivery of interventions for NTDs, rendering control and elimination efforts ineffective.
3. Provide a platform for the affected to share their experiences of living with the disability and document suggestions on how inclusive policy considerations can change the current status quo.
4. To devise strategies for sustaining the platform where the voices of the affected will continue to be amplified to bring positive impacts on the livelihood of the affected.
5. To synthesize the discussions into an inclusive actionable roadmap for the affected people, their health providers, policy makers, and researchers.

This report contributes to the fifth objective by outlining the roadmap created by workshop participants.

### Participants

**Fig 1:**
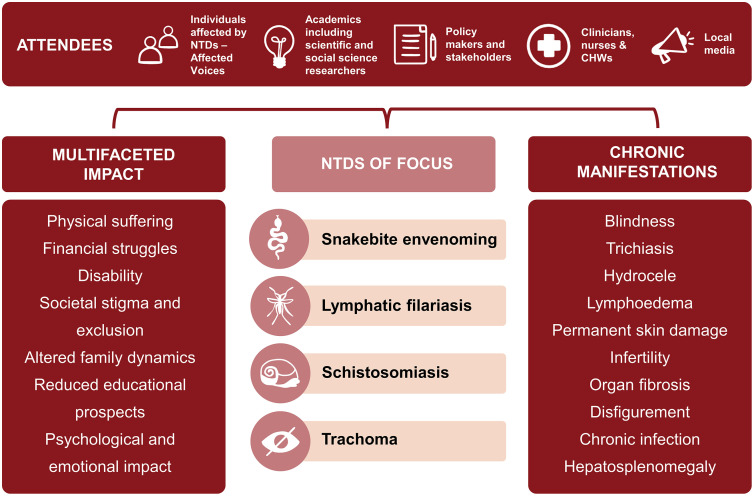
Workshop participants in Zimbabwe and Tanzania.

The participants for both workshops were from Tanzania; *n* = 37, male = 14 (age range 22–58 years) and female = 23 (age range 23–57 years); and Zimbabwe; *n* = 53, male = 18 (age range 11–62 years) and female = 34 (age range 13–65 years); and included affected voices (i.e., people living with the conditions or with lived experiences of the conditions associated with NTDs), the community health workers, community nurses, and specialist clinicians (e.g., gynecologists) providing health services for them, their ministry of health policy makers, and development partners. Participants from Zimbabwe and Tanzania attended both meetings in person. The affected voices participants aged between 11 and 65 years were victims of four NTDs detailed in Fig 1 and reported several chronic manifestations and multifaceted impacts of the conditions on their daily lives (Fig 1).

In both countries, the participants were predominantly farmers from NTD-endemic rural villages. In Zimbabwe, participants were predominately subsistence farmers growing maize, and keeping a few cattle, goats, and chickens. A few had family members working in the gold mines around Bindura and Shamva. Tanzanian participants were a combination of fishermen and subsistence farmers producing a variety of food crops while maintaining cattle and goats.

Also in attendance were researchers from the University of Edinburgh, Tanzania’s Catholic University of Health and Allied Sciences and National Institute for Medical Research, University of Zimbabwe and the Biomedical Research Institute in Sudan as well as local media.

### Focus group discussions and presentations

During the workshops, all participants present gave a short presentation on their lived experiences and responded to arising questions. Focus group discussions then followed the presentations. All workshop activities, including the presentations, were translated into Shona, Kiswahili, and English so that all participants could understand the deliberations. Their narratives provided poignant insights into their challenges, including reduced income, inability to carry out daily activities, stigma, health system experiences, and possible solutions for improving their care and support. Health workers presented their operational experiences of providing healthcare to the affected people while policy makers focused on gaps in NTD evidence and policy gaps. Finally, the researchers presented results from field, clinical, and situational analysis on currently available and accessible health services for NTD disease management. For example, it was clear that although problems such as female genital schistosomiasis, hepatic fibrosis, and ascites were common, health personnel lacked locally relevant technical and management guidelines.

### Roadmap synthesis

During the presentations, a designated pair of scribes (one from each country) summarized the main talking points from each person’s representation. On the last day in Tanzania, we applied thematic analysis [22] to workshop discussions to systematically identify key themes and translate them into clear, actionable points, facilitating the development of an actionable roadmap. Briefly, the thematic analysis followed a systematic six-stage process. First, transcripts from the scribes were read out to ensure familiarization with the data. Second, initial codes were generated by systematically highlighting and collating text segments into different themes; e.g., *“lack of diagnostics”, “lack of awareness among health workers”,* and *“clinic opening hours”* were clustered into the theme “systemic barriers”. Third, related codes were collated into potential themes that captured broader patterns across the dataset, in this case this fell under systemic issues. Fourth, these themes were reviewed by all participants and refined to ensure they accurately reflected the coded data and overall content. Fifth, all the participants then discussed actionable solutions to the challenges. Sixth, the summary solutions were collated for each theme, supported by illustrative quotations from participants, to present the findings in a concise and transparent manner. This information was used to produce the actionable roadmap detailed below.

## Results

### Co-created actionable roadmap

#### Call to action.

**Fig 2:**
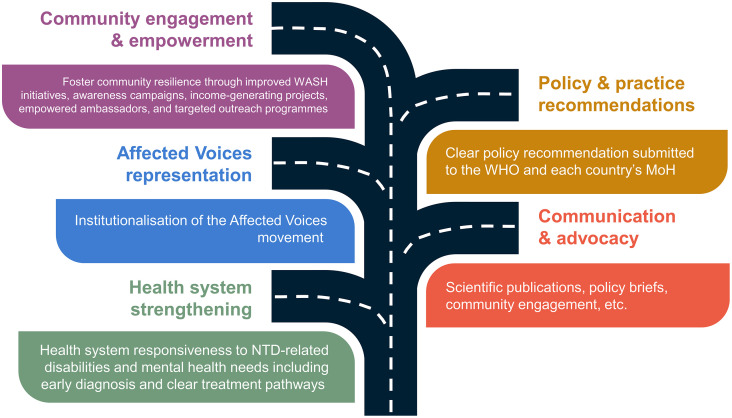
Roadmap call to action for NTD morbidity management.

The workshop participants made five calls to action for the affected voices, health workers, policy makers, ministries of health, and development partners. The thematic analysis identified five key themes; policy guidelines, communication and advocacy, community engagement and empowerment, affected voices representation, and health systems strengthening. The workshop participants summarized the main actions under each theme to form a roadmap for the improvement of the management of chronic NTDs (Fig 2).

**Fig 3:**
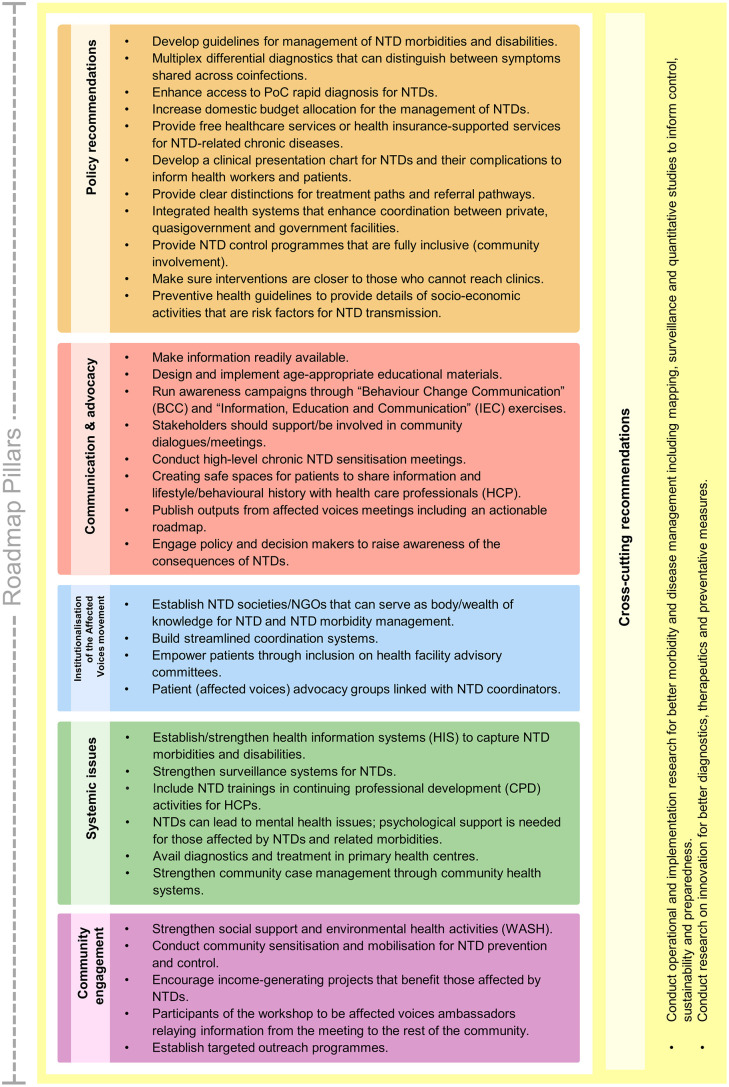
Roadmap actionable recommendations for NTD morbidity management.

Thereafter, the participants discussed actionable solutions to the challenges identified through the workshop presentations and focus group discussions. Through this, the workshop participants co-created inclusive actionable recommendations (Fig 3), detailing actions that need to be taken by the people affected by the diseases (sufferers and carers), community health workers, primary and tertiary health care professionals, ministry of health policy makers, researchers, and development partners. The actions ranged from those actionable immediately (e.g., producing this report and meeting policy brief, and participants becoming affected voices champions), to medium-and long-term tasks. The recommendations included two cross-cutting actions—i) conducting operational and implementation research for better morbidity and disease management, including mapping, surveillance and quantitative studies to inform control, sustainability, and preparedness; and ii) developing better diagnostics, especially multiplex point-of-care (POC) rapid diagnostics for differential diagnosis as well as therapeutics and preventative measures.

#### Gender aspects.

While both male and female adult participants did not readily seek medical interventions for early NTD symptoms for themselves, the focus group discussions indicated that the female participants, mostly mothers and grandmothers, were more likely to seek medical attention when it was their ward affected rather than for themselves. This was largely due to the female participants shouldering the child-caring burden and noticing symptoms early. The female participants also indicated that they discussed health among themselves more readily than the male participants.

## Discussion and conclusion

The participants living with chronic manifestations of NTDs recognized that they are currently among the most neglected of NTD sufferers. The desire and necessity for empowerment were expressed with the call for education to enable self-help and self-care, as well as representation and participation in NTD control decision-making forums. This workshop was the start of an affected voices movement for multiple NTDs to complement the few disease-specific affected voices movements already in existence, for e.g., leprosy [23] and noma [24].

The gender distribution of the participants of roughly one-third male and two-thirds female is partly reflective of the distribution of these diseases in the communities, highlighting the importance of considering gender in NTD morbidity management. A qualitative study in Kenya revealed that NTDs disproportionately affect women and girls due to gender-specific roles and responsibilities [13]. Similarly, a study in Ghana investigating how gender dynamics affect the management of skin-related NTDs such as Buruli ulcer, leprosy, and yaws found that women, despite having better knowledge about these diseases, often faced barriers to accessing treatment due to unequal power relations and limited access to resources [25]. The gender differences in health-seeking behaviors emphasized the need for gender-sensitive approaches in engaging the different genders to seek timely intervention. The WHO Secretariat’s Roadmap to Advance Gender Equality, Human Rights, and Health Equity (2023–2030) [26] already provides a comprehensive strategy for supporting Member States in implementing gender-responsive approaches, strengthening the respect for and protection of health-related human rights, and identifying and addressing health inequities through leadership, capacity building, accountability, tools, and technical assistance. It aims to embed these principles across planning, resource allocation, implementation, and reporting systems, coordinated by the Gender, Rights, and Equity Department within the WHO’s leadership structure. Ensuring that NTDs are specifically included in this will accelerate efforts to close the gender gaps in health access and health service availability for all. The participants valued a movement that was inclusive of both NTD sufferers and the diverse group of stakeholders involved. They recommended maintaining this inclusive format moving forward. Lived experiences give a more informative representation of the impact of NTDs on those affected, and as such the engagement and participation of NTD-affected people need to be fully inclusive across gender, age, and disease stages. Only two children aged 11 and 13, both from Zimbabwe, participated in the study. In the future, engagement of younger NTD sufferers will provide a wider picture of the NTD impacts on their lives, as well as potential for developing context-age-appropriate interventions.

The workshops were driven by a desire by the stakeholders in the full NTD ecosystem to accelerate progress in the disease management of chronic NTD conditions. As such, some actions have ensued from the recommendations made during the workshops. Firstly, the publication of this actionable roadmap was one of the action points recommended by the workshop participants in order to amplify the voices of affected people, as well as accelerate progress in improving the treatment, health services, and management of NTD chronic disease. Secondly, the policy brief developed from this roadmap was launched on World NTD Day 2025 and is now publicly available online [27]. Thirdly, engagement with ministries of health in Zimbabwe and Tanzania on the management of chronic NTD conditions has already commenced. Finally, a follow-on collaborative implementation project between Tanzania and Zimbabwe project has now commenced [28].
